# Reemergence of Enterovirus 71 Epidemic in Northern Taiwan, 2012

**DOI:** 10.1371/journal.pone.0116322

**Published:** 2015-03-16

**Authors:** Shu-Ting Luo, Pai-Shan Chiang, Wan-Yu Chung, Min-Yuan Chia, Kuo-Chien Tsao, Ying-Hsiang Wang, Tzou-Yien Lin, Min-Shi Lee

**Affiliations:** 1 National Institute of Infectious Disease and Vaccinology, National Health Research Institutes (NHRI), Zhunan Town, Miaoli County, Taiwan; 2 Department of Medical Biotechnology and Laboratory Science, College of Medicine, Chang Gung University, Gueishan Town, Taoyuan County, Taiwan; 3 Department of Laboratory Medicine, Linkou Chang Gung Memorial Hospital, Gueishan Town, Taoyuan county, Taiwan; 4 Department of Pediatrics, Chiayi Chang Gung Memorial Hospital, Puzi City, Chiayi County, Taiwan; 5 Department of Pediatrics, Linkou Chang Gung Memorial Hospital, Gueishan Town, Taoyuan County, Taiwan; 6 Graduate Institute of Clinical Medical Sciences, College of Medicine, Chang Gung University, Gueishan Town, Taoyuan County, Taiwan; University of Minnesota, UNITED STATES

## Abstract

**Background:**

Enterovirus 71 (EV71) belongs to picornavirus family and could be classified phylogenetically into three major genogroups (A, B and C) including 11 genotypes (A, B1-B5 and C1-C5). Since 1997, EV71 has caused large-scale of epidemics with neurological complications in Asian children. In Taiwan, nationwide EV71 epidemics with different predominant genotypes have occurred cyclically since 1998. A nationwide EV71 epidemic occurred again in 2012. We conducted genetic and antigenic characterizations of the 2012 epidemic.

**Methods:**

Chang Gung Memorial Hospital (CGMH) is a medical center in northern Taiwan. In CGMH, specimens were collected from pediatric inpatients with suspected enterovirus infections for virus isolation. Enterovirus isolates were serotyped and genotyped and sera from EV71 inpatients were collected for measuring neutralizing antibody titers.

**Results:**

There were 10, 16 and 99 EV71 inpatients identified in 2010, 2011 and 2012, respectively. There were 82 EV71 isolates genotyped, which identified 17 genotype C4a viruses and 65 genotype B5 viruses. The genotype B5 viruses were not detected until November 2011 and caused epidemics in 2012. Interestingly, the B5-2011 viruses were genetically distinguishable from the B5 viruses causing the 2008 epidemic and are likely introduced from China or Southeastern Asia. Based on antigenic analysis, minor antigenic variations were detected among the B5-2008, B5-2011, C4a-2008 and C4a-2012 viruses but these viruses antigenically differed from genotype A.

**Conclusions:**

Genotype B5 and C4a viruses antigenically differ from genotype A viruses which have disappeared globally for 30 years but have been detected in China since 2008. Enterovirus surveillance should monitor genetic and antigenic variations of EV71.

## Introduction

Enterovirus 71 (EV71) was first described in California, USA, in 1969. Since then, EV71 has been identified globally. The clinical spectrum of EV71 infection ranges from herpangina, hand-foot-mouth disease (HFMD) to severe cases with central nervous system (CNS) and cardiopulmonary involvements[1]. Follow-up studies further demonstrated that CNS-complicated EV71 infections could cause long-term cognitive and motor deficits[2, 3]. Globally, two patterns of EV71 outbreaks have been reported: small-scale outbreaks with low mortality and large-scale outbreaks with high mortality. The latter pattern occurred in Bulgaria in 1975, in Hungary in 1978, in Malaysia in 1997, in Taiwan in 1998, in Singapore in 2000, in Vietnam in 2005, in Brunei in 2006, in China since 2007, and recently in Cambodia in 2012[1, 4, 5]. Since the 1998 epidemic, Taiwan Centers for Disease Control has established national enterovirus surveillance system and EV71 has been detected as an endemic disease with cyclical nationwide epidemics every 3–4 years[1, 6, 7].

In response to the considerable public health concern worldwide due to the virulence of EV71, there has been intensified study of the phylogenetic relationships between EV71 isolates. Several regions of the EV71 genome have been used for phylogenetic analysis, the capsid protein VP1 is considered most robust for evolutionary study due to a high degree of diversity and lack of involvement in recombination[1]. Using this region for analysis, EV71 viruses are classified into three major genogroups (A, B, and C). Genogroup A disappeared in 1970s and reemerged in China in 2008; but genogroups B and C are widely circulating in Asia. Genogroups B and C can be further divided into genotypes B1–B5 and C1–C5, respectively and genotype C4 is classified into subgenotype C4a and C4b[1]. Recently, genogroups D, E and F were identified in India.[8] Interestingly, genotype replacements have been well documented in highly epidemic countries such as Malaysia, Vietnam and Taiwan[1]. In Taiwan, the predominant genotypes were C2 in 1998, B4 in 1999–2003, C4a in 2004–2005, and B5 in 2008–2009.[7] Because the phenomenon of genotype replacement could have critical implications for selection of vaccine strains, it needs to be well clarified[6, 7, 9, 10]. In 2012, a nationwide epidemic occurred again in Taiwan, which provided us with a unique opportunity to conduct genetic and antigenic analysis.

## Methods

### Study populations

Chang Gung Memorial Hospital (CGMH) is a medical center in northern Taiwan and was chosen as a study site because it has large pediatric populations and serves residents from rural and urban areas in northern Taiwan[10–12]. In CGMH, clinical specimens are routinely collected for virus isolation from hospitalized pediatric patients with suspected enterovirus infections (herpangina, HFMD, or nonspecific febrile illness during EV71 epidemics). Convalescent sera were also collected from EV71 patients for measuring neutralizing antibody titers. This study was approved by the CGMH Ethics Committee and written informed consents were obtained from guardians of participating children.

### Clinical and laboratory definitions

Evidence of herpangina included oral ulcerations on anterior tonsillar pillars, soft palate, buccal mucosa, or uvula. Evidence of HFMD included oral ulcers on the tongue and buccal mucosa and a vesicular rash on the hands, feet, knees, or buttocks. Nonspecific febrile illness was defined as a rectal temperature greater than 38°C without other symptoms. In complicated cases, aseptic meningitis was defined as a clinically compatible illness with cerebrospinal fluid pleocytosis (>5 leukocytes/mm^3^ in patients >1 month or >25 leukocytes/mm^3^ in neonates) and negative bacterial cultures. Encephalitis was characterized by an altered level of consciousness accompanied by cerebrospinal fluid pleocytosis. Evidence of a poliomyelitis-like syndrome included acute limb weakness with diminished reflexes and muscular strength. A diagnosis of encephalomyelitis was made when there was evidence of encephalitis and poliomyelitis-like syndrome. Evidence of pulmonary complications include pulmonary edema, hemorrhage or diffuse ground-glass infiltrations on roentgenography with decreased PaO2/FiO2 <300[13]. Acute heart failure was characterized by evidence of decreased contractility on echocardiography, arrhythmia, an enlarged heart, and elevations in cardiac enzymes that are markers for cardiac damage[14]. Laboratory evidence of EV71 infection was defined as the isolation of EV71 from a throat swab, a rectal swab, or a stool sample.

### Virologic analysis

In CGMH, clinical samples (throat swabs, rectal swabs, or stool samples) were routinely collected for virus isolation from pediatric inpatients with suspected enterovirus infections (herpangina, HFMD, or nonspecific febrile illness during enterovirus epidemics). Samples were inoculated into human embryonic fibroblast, LLC-MK2, HEp-2, and rhabdomyosarcoma (RD) cell cultures. When enteroviral cytopathic effect involved more than 50% of the cell monolayer, cells were scraped and subjected to indirect fluorescent antibody staining with enteroviruses monoclonal antibodies[15]. VP1 genes of isolated EV71 viruses were sequenced and genotyped by phylogenetic analysis using the Neighbor-joining method in MEGA 4 software as described previously[10, 16]. It is not cost-effective to conduct complete VP1 sequencing for all isolates. Therefore, we used the following selection criteria for virus typing: 1) all isolates were genotyped if monthly number of EV71 isolates was less than 5; 2) at least 5 isolates were genotyped if monthly number of isolates was 5~10; and 3) about 40% of isolates were genotyped if monthly number of isolates was >10. Backgrounds of reference virus sequences used in the phylogenetic analysis were listed in S1 Table. Sequences of those EV71 strains generated in this study have been submitted to GenBank (accession codes KF154285-KF154347, KF154349-KF154356, KF933353, KP113681-KP113692).

### Serologic assays

Laboratory methods for measuring EV71 serum neutralizing antibody titers followed standard protocols[17]. Twofold serially diluted sera (1:8–1:512) and virus working solution containing 100 TCID_50_ of EV71 were mixed on 96-well microplates and incubated with RD cells. A cytopathic effect was observed in a monitor linked with an inverted microscope after an incubation period of 4 to 5 days. The neutralization titers were read as the highest dilution that could result in a 50% reduction in the cytopathic effect. Each test sample was run simultaneously with cell control, serum control, and virus back titration. The starting dilution was 1:8; the cutoff level of seropositivity was set at 8. Undetectable titer was assigned a level of 2 for the calculation of geometric mean titer (GMT).

### Statistical analysis

Neutralization antibody titers were log-transformed to calculate the GMT and 95% confidence interval (95% CI). The statistical association between two nominal or ordinal variables was tested by the χ^2^ test or Fisher’s Exact test as appropriate. All statistical analyses were performed using Microsoft Excel (Microsoft, Redmond, WA, USA) or SAS (SAS Institutes, Cary, NC, USA).

## Results

As shown in Table 1, there were 10, 16 and 99 EV71 inpatients identified in 2010, 2011 and 2012, respectively. Among the 125 EV71 inpatients, 23 (18.4%) developed neurological complications. Age distributions of the 102 uncomplicated and 23 complicated EV71 patients were 59.8% and 52.2% for ≦3 years of age, 21.6% and 39.1% for 4–6 years of age, and 18.6% and 9.1% for ≧7 years of age, respectively. Proportions of the complicated EV71 inpatients in these 3 years were 10.0% (95% CI 0–31.5%), 25.0% (95% CI 1.9–48.1%) and 18.2% (95% CI 6.5–29.9%) (Table 1). In total, 82 EV71 isolates were genotyped based on genetic analysis of complete VP1 genes, which identified 17 C4a genotypes and 65 B5 genotypes. Proportions of complicated infections were 29.4% (5/17) (95% CI 6–52.8%) for C4a genotype and 16.9% (11/65) (95% CI: 7.6–26.3%) for B5 genotype without statistical significance.

**Table 1:** Number of EV71 inpatients and virus genotypes identified in a medical center in Northern Taiwan, 2010–2012.

Variables	2010	2011	2012	Total
No. of EV71 inpatients (complicated cases)	10 (1)	16 (4)	99 (18)	125 (23)
%(95% CI) of EV71 inpatients with complications	10.0 (0–31.5)	25.0 (1.9–48.1)	18.2 (6.5–29.9)	18.4 (11.5–25.3)
No. of EV71 isolates sequenced (genotype)	10 (10 C4a)	16 (11 B5, 5 C4a)	56 (54 B5, 2 C4a)	82 (65 B5, 17 C4a)
No. of post-infection serum collected (genotype)	4 (4 C4a)	10 (5 B5, 5 C4a)	16 (15 B5, 1 C4a)	30 (20 B5, 10 C4a)

**Fig 1:**
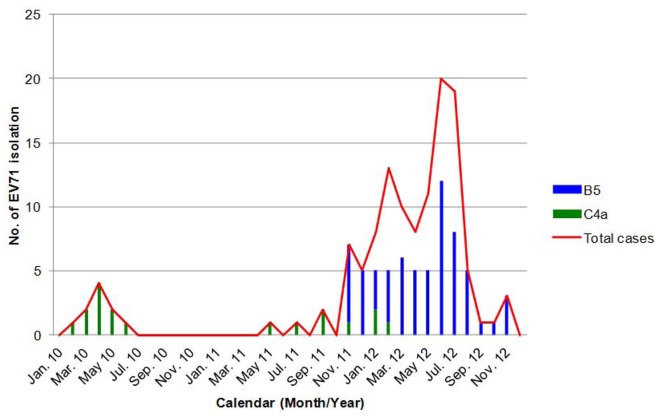
Distribution of enterovirus 71 (EV71) genotypes (B5 and C4a) isolated in pediatric inpatients in a medical center in northern Taiwan, 2010~2012.

**Fig 2:**
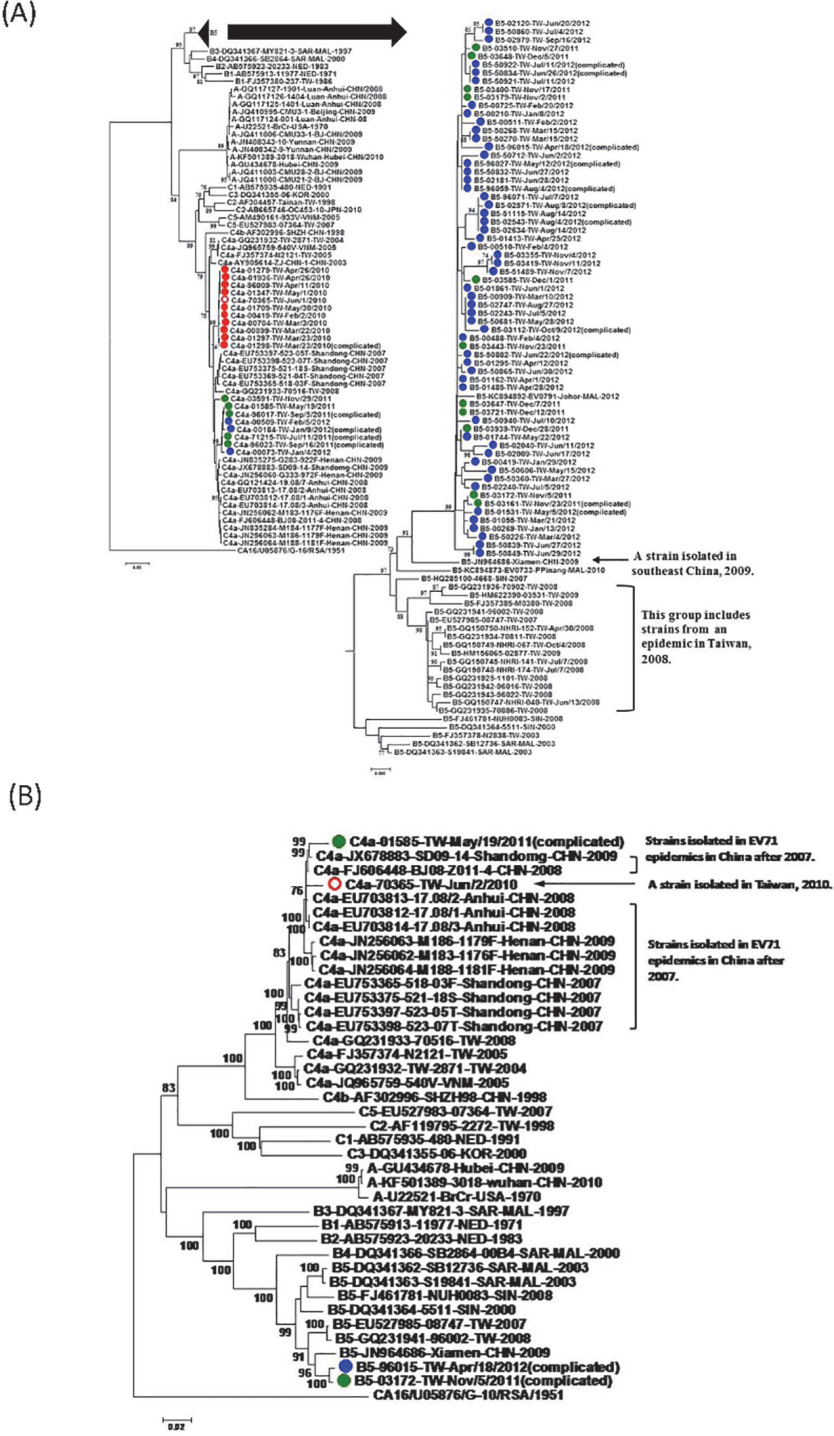
Phylogenetic analysis of VP1 gene (A) and full genomes (B) of EV71 virus. The phylogenetic trees were generated by the neighbor-joining method with 1000 bootstraps for EV71 isolates from this study and other published EV71 strains based on VP1 gene (891bp) and complete genome. Only bootstrap values over 70% were shown. Coxsackievirus A16 was used as the outlier. Sequences of EV71 strains isolated from this study in 2010, 2011 and 2012 were labeled with red, green and blue dots, respectively. A sequence of EV71 isolate from an outpatient in 2010 was labeled with red circle. Sequences of those EV71 strains generated in this study have been submitted to GenBank (accession codes KF154285-KF154347, KF154349-KF154356, KF933353, KP113681-KP113692).

Based on monthly distribution of the 82 EV71 isolates, the predominant genotype isolated from January 2010 to September 2011 belonged to C4a genotype but shifted to B5 genotype after October 2011. The genotype B5 viruses continued to circulate and reached peak in June 2012 (Fig. 1). Interestingly, the 65 genotype B5 viruses isolated in 2011−2012 are highly related (nucleotide identity >98%) and phylogenetically more similar to the B5 virus isolated in Xiamen, China in 2009 than to the B5 viruses circulating in Taiwan in 2007–2009 (Fig. 2A). In addition, a Malaysia strain (B5-KC894892-EV0791-Johor-MAL-2012) was also grouped together with the Taiwan B5-2011 strains. Due to limited sequence data available from Southeastern Asia in 2009–2011, we could not exclude that other B5-Xiamen-2009-like viruses also circulated in Southeastern Asia before 2011. Complete genome of two genotype B5 viruses isolated in Taiwan in 2011 and 2012 were further sequenced and complete genome analysis confirmed the relationship (Fig. 2B). Overall, the phylogenetic data indicate the potential international spreading of genotype B5 viruses from China or Southeastern Asia to Taiwan in 2011. Surprisingly, genotype C4a viruses isolated in 2010 and 2011–2012 were genetically differentiable and they were likely introduced to Taiwan separately based on phylogenetic analysis of VP1 genes and complete genomes (Fig. 2A, 2B).

To elucidate the antigenic relationship among the genotype C4a and B5 viruses isolated in Taiwan, we collected 30 post-infection children sera including 20 infected with genotype B5 and 10 infected with genotype C4a to measure cross-reactive neutralization antibody titers against 5 EV71 strains (one genotype A virus, two genotype C4a viruses and two genotype B5 viruses) (Table 2). As shown in Table 2, the 20 children infected with B5 viruses have significant lower neutralizing antibody titers against genotype A virus (GMT 66, 95% CI 41–107) and genotype C4a-2012 viruses (GMT 94, 95% CI 58–151) than B5–2008 (GMT 350, 95% CI 190–643). In the 10 children infected with genotype C4a viruses, serum neutralizing antibody titers against genotype A virus was the lowest among the cross-reactive neutralizing antibody titers against 5 virus strains but the differences did not reach statistical significance due to small sample size. Overall, genotype A virus antigenically differs from genotype B5 and C4a viruses but antigenic variations between genotype B5 and C4a viruses are not clear-cut.

**Table 2:** Cross-reactive neutralizing antibody titers in children infected with EV71 C4a and B5 genotypes.

Virus strains tested	Children infected with genotype B5 (n = 20) GMT (95% CI)	Children infected with Genotype C4a (n = 10) GMT (95% CI)
A-BrCr-USA-70	66 (41–107)	60 (25–145)
B5-NHRI141-TW-08	350 (190–643)	315 (108–921)
B5–3172-TW-11	163 (100–265)	147 (56–383)
C4a-70516-TW-08	142 (92–220)	223 (97–514)
C4a-00184-TW-12	94 (58–151)	194 (83–454)

## Discussion

EV71 viruses are widely circulating in Asian countries and genotype replacements have been well documented in Malaysia, Vietnam and Taiwan[6, 18–20]. In Taiwan, the predominant EV71 genotypes were C2 in 1998, B4 in 2000–2003, C4a in 2004–2005, and B5 in 2008–2009. Interestingly, 15 sporadic genotype C4a cases were detected in 2010 and 2011 but EV71 epidemics did not occur until genotype B5 viruses were introduced to Taiwan after the middle of 2011, which is similar to data collected in central Taiwan and national surveillance data collected by Taiwan Centers for Disease Control [21, 22].

The potential reasons for the genotype replacement observed in this study include: 1) significant antigenic variations exist among the B5-2008, C4a-2008, C4a-2012 and B5-2012 viruses; 2) the B5-2012 viruses may have higher transmission efficiency than the C4a-2012 viruses; 3) other unknown biological or epidemiological mechanism. Based on antigenic analysis using post-infection children sera in this study, genotype A virus antigenically differs from genotype B5 and C4a viruses but antigenic variations between genotype B5 and C4a viruses are not clear-cut, which are similar to other clinical studies[7, 20, 21, 23]. Regarding the transmission efficiency of the B5-2012 and C4a-2010 viruses, no relevant animal model is available to evaluate transmission efficiency of enterovirus 71. Longitudinal studies and animal models are desirable to clarify the mechanism of EV71 genotype replacement.

Interestingly, the genotype A viruses have disappeared globally for 30 years since 1970s but have been detected in many parts of China (Anhui, Beijing, Hubei, and Yunnan) since 2008 (Fig. 2A)[24–26]. Source of the reemergence has not been well-defined and would require genomic studies to clarify. Moreover, clinical and epidemiological significances of the observed antigenic variations between genotype A and other genogroup EV71 viruses are critical to vaccine development and would need longitudinal studies to clarify.

International spreading of EV71 is common and may play an important role for reemergence of large-scale epidemics in Asia.[1] For example, EV71 genotype B5 viruses spread from Southeastern Asia to Taiwan in 2007 and caused a nation-wide epidemic in 2008[10, 27]. In 2011, EV71 genotype C4a viruses spread from China to Vietnam and caused a large-scale epidemic in HCM City and southern Vietnam[28]. Recently, Western Pacific Regional Office of the World Health Organization has established a HFMD reporting system to collect monthly HFMD case number from China, Hong Kong, Macao, Japan, Republic of Korea, Singapore and Vietnam (http://www.wpro.who.int). However, the reporting systems vary from country to country and do not include virus identification data, which make international comparisons impossible. As learnt from global influenza surveillance system, an international enterovirus surveillance system with harmonized clinical definitions and virus identification data is urgently desirable to understand epidemiology and disease burden of EV71 in Asia.

In conclusion, the predominant viruses in the 2011−2012 epidemic in Taiwan belong to genotype B5 and are phylogenetically close to the B5 viruses circulating in Xiamen, China in 2009. Genotype B5 and C4a viruses antigenically differ from genotype A viruses which have disappeared for 30 years but have reemerged in China since 2008. Enterovirus surveillance and development of EV71 vaccines should monitor genetic and antigenic variations of enterovirus 71 through international collaborations.

## Supporting Information

S1 Table List of reference virus strains used to conduct phylogenetic analysis of full genome and VP1 sequences. (DOC)

## References

[1] Lee MS, Tseng FC, Wang JR, Chi CY, Chong P, et al (2012) Challenges to licensure of enterovirus 71 vaccines. PLoS Negl Trop Dis 6(8): e1737 10.1371/journal.pntd.0001737 22953003 PMC3429393

[2] Huang MC, Wang SM, Hsu YW, Lin HC, Chi CY, et al (2006) Long-term cognitive and motor deficits after enterovirus 71 brainstem encephalitis in children. Pediatrics 118(6): e1785–1788. 17116698 10.1542/peds.2006-1547

[3] Chang LY, Huang LM, Gau SS, Wu YY, Hsia SH, et al (2007) Neurodevelopment and cognition in children after enterovirus 71 infection. N Engl J Med 356(12): 1226–1234. 17377160 10.1056/NEJMoa065954

[4] World Health Organization (2011) A Guide to Clinical Management and Public Health Response for Hand, Foot and Mouth Disease (HFMD). Geneva: WHO.

[5] Seiff A (2012) Cambodia unravels cause of mystery illness. Lancet 380(9838): 206 22826834 10.1016/s0140-6736(12)61200-8

[6] Chia MY, Chiang PS, Chung WY, Luo ST, Lee MS (2013) Epidemiology of Enterovirus 71 Infections in Taiwan. Pediatr Neonatol. 10.1016/j.pedneo.2013.07.007 24120535

[7] Huang ML, Chiang PS, Chia MY, Luo ST, Chang LY, et al (2013) Cross-reactive neutralizing antibody responses to enterovirus 71 infections in young children: implications for vaccine development. PLoS Negl Trop Dis 7(2): e2067 10.1371/journal.pntd.0002067 23459633 PMC3573098

[8] Rao CD, Yergolkar P, Shankarappa KS (2012) Antigenic diversity of enteroviruses associated with nonpolio acute flaccid paralysis, India, 2007–2009. Emerg Infect Dis 18(11): 1833–1840. 10.3201/eid1811.111457 23092622 PMC3559176

[9] Fan X, Jiang J, Liu Y, Huang X, Wang P, et al (2013) Detection of human enterovirus 71 and Coxsackievirus A16 in an outbreak of hand, foot, and mouth disease in Henan Province, China in 2009. Virus Genes 46(1): 1–9. 10.1007/s11262-012-0814-x 23080402

[10] Lee MS, Lin TY, Chiang PS, Li WC, Luo ST, et al (2010) An investigation of epidemic enterovirus 71 infection in Taiwan, 2008: clinical, virologic, and serologic features. Pediatr Infect Dis J 29(11): 1030–1034. 10.1097/INF.0b013e3181e52945 20543760

[11] Luo ST, Chiang PS, Chao AS, Liou GY, Lin R, et al (2009) Enterovirus 71 maternal antibodies in infants, Taiwan. Emerg Infect Dis 15(4): 581–584. 10.3201/eid1504.081550 19331737 PMC2671432

[12] Lee MS, Chiang PS, Luo ST, Huang ML, Liou GY, et al (2012) Incidence rates of enterovirus 71 infections in young children during a nationwide epidemic in Taiwan, 2008–09. PLoS Negl Trop Dis 6(2): e1476 10.1371/journal.pntd.0001476 22348156 PMC3279337

[13] Chang LY, Tsao KC, Hsia SH, Shih SR, Huang CG, et al (2004) Transmission and clinical features of enterovirus 71 infections in household contacts in Taiwan. Jama 291(2): 222–227. 14722149 10.1001/jama.291.2.222

[14] Ho M, Chen ER, Hsu KH, Twu SJ, Chen KT, et al (1999) An epidemic of enterovirus 71 infection in Taiwan. Taiwan Enterovirus Epidemic Working Group. N Engl J Med 341(13): 929–935. 10498487 10.1056/NEJM199909233411301

[15] Lin TL, Li YS, Huang CW, Hsu CC, Wu HS, et al (2008) Rapid and highly sensitive coxsackievirus a indirect immunofluorescence assay typing kit for enterovirus serotyping. J Clin Microbiol 46(2): 785–788. 18032614 10.1128/JCM.01114-07 PMC2238120

[16] Tamura K, Dudley J, Nei M, Kumar S (2007) MEGA4: Molecular Evolutionary Genetics Analysis (MEGA) software version 4.0. Mol Biol Evol 24(8): 1596–1599. 17488738 10.1093/molbev/msm092

[17] Huang ML, Chiang PS, Luo ST, Liou GY, Lee MS (2010) Development of a high-throughput assay for measuring serum neutralizing antibody against enterovirus 71. J Virol Methods 165(1): 42–45. 10.1016/j.jviromet.2009.12.015 20036286

[18] Chua KB, Chua BH, Lee CS, Chem YK, Ismail N, et al (2007) Genetic diversity of enterovirus 71 isolated from cases of hand, foot and mouth disease in the 1997, 2000 and 2005 outbreaks, Peninsular Malaysia. Malays J Pathol 29(2): 69–78. 19108398

[19] Ooi MH, Wong SC, Podin Y, Akin W, del Sel S, et al (2007) Human enterovirus 71 disease in Sarawak, Malaysia: a prospective clinical, virological, and molecular epidemiological study. Clin Infect Dis 44(5): 646–656. 17278054 10.1086/511073

[20] Thoa le PK, Chiang PS, Khanh TH, Luo ST, Dan TN, et al (2013) Genetic and antigenic characterization of enterovirus 71 in Ho Chi Minh City, Vietnam, 2011. PLoS One 8(7): e69895 10.1371/journal.pone.0069895 23922846 PMC3726754

[21] Huang YP, Lin TL, Lin TH, Wu HS (2013) Antigenic and genetic diversity of human enterovirus 71 from 2009 to 2012, taiwan. PLoS One 8(11): e80942 10.1371/journal.pone.0080942 24348916 PMC3858369

[22] Wu WH, Kuo TC, Lin YT, Huang SW, Liu HF, et al (2013) Molecular epidemiology of enterovirus 71 infection in the central region of Taiwan from 2002 to 2012. PloS one 8(12): e83711 10.1371/journal.pone.0083711 24391812 PMC3877097

[23] Huang YP, Lin TL, Hsu LC, Chen YJ, Tseng YH, et al (2010) Genetic diversity and C2-like subgenogroup strains of enterovirus 71, Taiwan, 2008. Virol J 7: 277 10.1186/1743-422X-7-277 20959020 PMC2975644

[24] Yu H, Chen W, Chang H, Tang R, Zhao J, et al (2010) Genetic analysis of the VP1 region of enterovirus 71 reveals the emergence of genotype A in central China in 2008. Virus Genes 41(1): 1–4. 10.1007/s11262-010-0472-9 20306124

[25] Yang Z, Lu S, Xian J, Ye J, Xiao L, et al (2013) Complete genome sequence of a human enterovirus 71 strain isolated in wuhan, china, in 2010. Genome Announc 1(6). 10.1128/genomeA.01112-13 PMC3873616 24371206

[26] Zhu J, Luo Z, Wang J, Xu Z, Chen H, et al (2013) Phylogenetic analysis of Enterovirus 71 circulating in Beijing, China from 2007 to 2009. PLoS One 8(2): e56318 10.1371/journal.pone.0056318 23418551 PMC3572022

[27] Huang SW, Hsu YW, Smith DJ, Kiang D, Tsai HP, et al (2009) Reemergence of enterovirus 71 in 2008 in taiwan: dynamics of genetic and antigenic evolution from 1998 to 2008. J Clin Microbiol 47(11): 3653–3662. 10.1128/JCM.00630-09 19776232 PMC2772620

[28] Khanh TH, Sabanathan S, Thanh TT, Thoa le PK, Thuong TC, et al (2012) Enterovirus 71-associated hand, foot, and mouth disease, Southern Vietnam, 2011. Emerg Infect Dis 18(12): 2002–2005. 10.3201/eid1812.120929 23194699 PMC3557876

