# Supplementary material for: Reemergence of Enterovirus 71 Epidemic in Northern Taiwan, 2012
Source: PLoS One. 2015 Mar 16;10(3):e0116322. doi: 10.1371/journal.pone.0116322 (PMC4361668; doi:10.1371/journal.pone.0116322)
Supplement: S1 Table — (DOC) [file pone.0116322.s001.doc]

**Table S1. List of reference virus strains used to conduct phylogenetic analysis of full genome and VP1 sequences.**

| Virus ID | Accession no. | Genotype | Gene | Year | Country |
| --- | --- | --- | --- | --- | --- |
| BrCr-CA-70 | U22521 | A | Full genome | 1970 | USA |
| 001-Luan | GQ117124 | A | VP1 | 2008 | China |
| 1901-Luan | GQ117127 | A | VP1 | 2008 | China |
| 1401-Luan | GQ117125 | A | VP1 | 2008 | China |
| 1404-Luan | GQ117126 | A | VP1 | 2008 | China |
| Hubei | GU434678 | A | Full genome | 2009 | China |
| CMU3-1 | JQ410995 | A | VP1 | 2009 | China |
| CMU21-2 | JQ411000 | A | VP1 | 2009 | China |
| CMU28-2 | JQ411003 | A | VP1 | 2009 | China |
| CMU33-1 | JQ411006 | A | VP1 | 2009 | China |
| 9-Yunnan | JN408342 | A | VP1 | 2009 | China |
| 10-Yunnan | JN408343 | A | VP1 | 2009 | China |
| 3018-Wuhan | KF501389 | A | Full genome | 2010 | China |
| 11977 | AB575913 | B1 | Full genome | 1971 | Netherland |
| 237 | EJ357380 | B1 | VP1 | 1986 | Taiwan |
| 20233 | AB575923 | B2 | Full genome | 1983 | Netherland |
| MY821-3 | DQ341367 | B3 | Full genome | 1997 | Malaysia |
| SB2864-SAR-00 | DQ341366 | B4 | Full genome | 2000 | Malaysia |
| 5511 | DQ341364 | B5 | Full genome | 2000 | Singapore |
| SB12736 | DQ341362 | B5 | Full genome | 2003 | Malaysia |
| S19841 | DQ341363 | B5 | Full genome | 2003 | Malaysia |
| N2838 | FJ357378 | B5 | VP1 | 2003 | Taiwan |
| 08747 | EU527985 | B5 | Full genome | 2007 | Taiwan |
| 4668 | HQ285100 | B5 | VP1 | 2007 | Singapore |
| NHRI-040 | GQ150747 | B5 | VP1 | 2008 | Taiwan |
| NHRI-067 | GQ150749 | B5 | VP1 | 2008 | Taiwan |
| NHRI-141 | GQ150745 | B5 | VP1 | 2008 | Taiwan |
| NHRI-152 | GQ150750 | B5 | VP1 | 2008 | Taiwan |
| NHRI-174 | GQ150748 | B5 | VP1 | 2008 | Taiwan |
| 96002 | GQ231941 | B5 | Full genome | 2008 | Taiwan |
| NUH0083 | FJ461781 | B5 | Full genome | 2008 | Singapore |
| 70902 | GQ231936 | B5 | VP1 | 2008 | Taiwan |
| M0380 | FJ357385 | B5 | VP1 | 2008 | Taiwan |
| 96002 | GQ231941 | B5 | VP1 | 2008 | Taiwan |
| 1101 | GQ231925 | B5 | VP1 | 2008 | Taiwan |
| 96016 | GQ231942 | B5 | VP1 | 2008 | Taiwan |
| 96022 | GQ231943 | B5 | VP1 | 2008 | Taiwan |
| 70811 | GQ231934 | B5 | VP1 | 2008 | Taiwan |
| 70886 | GQ231935 | B5 | VP1 | 2008 | Taiwan |
| Xiamen | JN964686 | B5 | Full genome | 2009 | China |
| 03531 | HM622390 | B5 | VP1 | 2009 | Taiwan |
| 02877 | HM156065 | B5 | VP1 | 2009 | Taiwan |
| EV0733 | KC894873 | B5 | VP1 | 2010 | Malaysia |
| EV0791 | KC894892 | B5 | VP1 | 2012 | Malaysia |
| 480 | AB575935 | C1 | Full genome | 1991 | Netherland |
| 2272 | AF119795 | C2 | Full genome | 1998 | Taiwan |
| Tainan | AF304457 | C2 | VP1 | 1998 | Taiwan |
| OC453-10 | AB665746 | C2 | VP1 | 2010 | Japan |
| 6 | DQ341355 | C3 | Full genome | 2000 | Korea |
| ZJ-CHN-1 | AY905614 | C4a | VP1 | 2003 | China |
| TW-2871 | GQ231932 | C4a | Full genome | 2004 | Taiwan |
| N2121 | FJ357374 | C4a | Full genome | 2005 | Taiwan |
| 540V | JQ965759 | C4a | Full genome | 2005 | Vietnam |
| 521-04T | EU753369 | C4a | VP1 | 2007 | China |
| 518-03F | EU753365 | C4a | Full genome | 2007 | China |
| 521-18S | EU753375 | C4a | Full genome | 2007 | China |
| 523-05T | EU753397 | C4a | Full genome | 2007 | China |
| 523-07T | EU753398 | C4a | Full genome | 2007 | China |
| 70516 | GQ231933 | C4a | VP1 | 2008 | Taiwan |
| 17.08/1 | EU703812 | C4a | Full genome | 2008 | China |
| 17.08/2 | EU703813 | C4a | Full genome | 2008 | China |
| 17.08/3 | EU703814 | C4a | Full genome | 2008 | China |
| 19.08/7 | GQ121424 | C4a | VP1 | 2008 | China |
| M183-1176F | JN256062 | C4a | Full genome | 2009 | China |
| M184-1177F | JN835284 | C4a | VP1 | 2009 | China |
| M186-1179F | JN256063 | C4a | Full genome | 2009 | China |
| M188-1181F | JN256064 | C4a | Full genome | 2009 | China |
| G283-922F | JN835275 | C4a | VP1 | 2009 | China |
| G333-972F | JN256060 | C4a | VP1 | 2009 | China |
| SHZH98 | AF302996 | C4b | Full genome | 1998 | China |
| 933V | AM490161 | C5 | VP1 | 2005 | Vietnam |
| 07364 | EU527983 | C5 | Full genome | 2007 | Taiwan |
| G-10 | U05876 | CVA-16 | Full genome | 1951 | RSA^a^ |

^a^RSA:Republic of South Africa
